# Emotion and location cues bias conceptual retrieval in people with deficient semantic control

**DOI:** 10.1016/j.neuropsychologia.2019.05.030

**Published:** 2019-08

**Authors:** Lucilla Lanzoni, Hannah Thompson, Danai Beintari, Katrina Berwick, Harriet Demnitz-King, Hannah Raspin, Maria Taha, Sara Stampacchia, Jonathan Smallwood, Elizabeth Jefferies

**Affiliations:** a Department of Psychology, University of York, UK; b School of Psychology, University of Surrey, UK; c Faculty of Brain Sciences, University College London, UK

**Keywords:** Stroke, Aphasia, Context, Cueing, Semantic, Spatial, Emotion

## Abstract

Visuo-spatial context and emotional valence are powerful cues to episodic retrieval, but the contribution of these inputs to semantic cognition has not been widely investigated. We examined the impact of visuo-spatial, facial emotion and prosody cues and miscues on the retrieval of dominant and subordinate meanings of ambiguous words. Cue photographs provided relevant visuo-spatial or emotional information, consistent with the interpretation of the ambiguous word being probed, while miscues were consistent with an alternative interpretation. We compared the impact of these cues in healthy controls and semantic aphasia patients with deficient control over semantic retrieval following left-hemisphere stroke. Patients showed greater deficits in retrieving the subordinate meanings of ambiguous words, and stronger effects of cueing and miscuing relative to healthy controls. These findings suggest that contextual cues that guide retrieval to the appropriate semantic information reduce the need to constrain semantic retrieval internally, while miscues that are not aligned with the task increase the need for semantic control. Moreover, both valence and visuo-spatial context can prime particular semantic interpretations, in line with theoretical frameworks that argue meaning is computed through the integration of these features. In semantic aphasia, residual comprehension relies heavily on facial expressions and visuospatial cues. This has important implications for patients, their families and clinicians when developing new or more effective modes of communication.

## Highlights

• Contextual information constraints semantic retrieval in stroke aphasia.
• Misleading emotional and spatial cues increase the need for semantic control.
• Findings have potential to facilitate communication in patients with aphasia.

## 1 Introduction

Although we retain a wealth of information about any given concept, only a subset of this information is relevant in a particular context (Jefferies, 2013; Schoen, 1988; Yee and Thompson-Schill, 2016). Sometimes, distant associations or less dominant aspects of knowledge are required to achieve a certain goal: we can readily identify that a rolled up newspaper can squash a fly, even though newspapers are normally associated with reading (Corbett et al., 2011; Jefferies, 2013). This semantic flexibility, reflecting the retrieval of non-dominant elements of concepts in a context-dependent manner, is thought to require semantic control processes that are separate from the conceptual store (Jefferies, 2013; Lambon Ralph et al., 2016; Thompson-Schill et al., 1997; Wagner et al., 2001; Whitney et al., 2011). According to the ‘hub and spoke’ account of semantic cognition (Lambon Ralph et al., 2016; Patterson et al., 2007), modality-specific features (‘spokes’) are integrated to form heteromodal conceptual representations within a ‘hub’ in the ventral anterior temporal lobes (ATL). When the pattern of semantic retrieval required by a task for a specific concept is aligned closely with its dominant features and associations within the semantic store, hub-spoke interactions should readily generate coherent semantic activation that can drive an appropriate response relatively automatically. However, when the most accessible information pertaining to a concept is *not* relevant (for example, when we use newspapers to swat flies), unconstrained semantic activation is less helpful. Accordingly, it is assumed that in these situations, semantic control mechanisms come into play, allowing us to produce flexible patterns of retrieval (Controlled Semantic Cognition account; Jefferies, 2013; Lambon Ralph et al., 2016).

This semantic flexibility is compromised in patients with semantic aphasia (SA) following left-hemisphere inferior frontal and/or temporoparietal stroke (Lambon Ralph et al., 2016; Noonan et al., 2013a; Noonan et al., 2010). Patients with SA have deregulated semantic cognition in both verbal and non-verbal tasks (Corbett et al., 2009a, 2009b; Gardner et al., 2012; Jefferies et al., 2008a; Jefferies and Lambon Ralph, 2006; Thompson et al., 2015). They have difficulty selecting targets in the presence of distractors with related meanings and show poorer comprehension of non-dominant interpretations of ambiguous words (e.g. when matching fire with rifle, as opposed to matching fire with hot; Noonan et al., 2010). Critically, these patients show inconsistent performance when the same concepts are probed under different cognitive demands, often performing the best in more constrained tasks in which semantic retrieval is strongly guided by the task itself (Jefferies and Lambon Ralph, 2006; Noonan et al., 2013a; Rogers et al., 2015). For example, Corbett et al. (2011) found that performance in a naturalistic task involving demonstrating the use of an object was significantly improved when SA patients were provided with the actual object (e.g. a hammer) and a picture of the usual recipient (e.g. a nail) compared to when they were verbally instructed to mime the use of the object (e.g. ‘show me how you would use a hammer). The original definition of semantic aphasia provided by Henry Head (1926) and Luria (1973) referred to a cluster of high-level interpretative deficits across modalities involving processing relationships between concepts. In this study and in previous publications by this group we have used the term semantic aphasia to refer to patients with multimodal semantic problems affecting both words and pictures. Other researchers using this term (e.g. Dragoy et al., 2017), have focused on problems at the sentence level, highlighting the difficulties of their SA cases with logical-grammatical structures and figurative speech. These sets of patients are likely to have overlapping deficits, although the cases reported here and by other studies from our group typically have some degree of impairment for single items, and therefore may have more severe heteromodal deficits of semantic cognition. Overall, this pattern of impairment is qualitatively distinct from deficits in semantic dementia: although both groups have multimodal semantic comprehension impairment affecting both verbal and non-verbal comprehension, semantic dementia gives rise to a gradual degradation of conceptual knowledge that is highly predictable across tasks, following atrophy and hypometabolism focused on the ventral ATL (Desgranges et al., 2007; Diehl et al., 2004; Mion et al., 2010; Mummery et al., 2000; Rosen et al., 2002; Studholme et al., 2004).

This neuropsychological evidence suggests that distinct neurocognitive components support conceptual representation and control, with left inferior frontal gyrus (IFG) and posterior middle temporal gyrus (pMTG) – regions commonly damaged in SA patients – critical for semantic control. Convergent evidence is provided by neuroimaging (Badre et al., 2005; Davey et al., 2015b; Davey et al., 2016; Noonan et al., 2013b; Thompson-Schill et al., 1997) and brain stimulation studies of healthy participants (Davey et al., 2015a Hallam et al., 2016; Hoffman et al., 2010; Whitney et al., 2011). These regions commonly activate across a wide range of semantic control manipulations – including for weak vs. Strong associations, decisions in the face of strong distractors and for ambiguous words, when there is a need to resolve competition between alternative interpretations (Bedny et al., 2008; Rodd et al., 2005; Vitello and Rodd, 2015; Vitello et al., 2014; Zempleni et al., 2007). Inhibitory TMS delivered to left IFG and pMTG elicits equal disruption of tasks requiring semantic control, while there is no effect on either easier semantic judgements or non-semantic decisions (Davey et al., 2015a Hoffman et al., 2010; Whitney et al., 2011). Left IFG and pMTG show a response to semantic control manipulations across modalities (Krieger-Redwood et al., 2015) and are largely distinct from multiple-demand regions that support domain-general cognitive control (Davey et al., 2016; Noonan et al., 2013b). As these aspects of control occupy adjacent regions along the cortical surface (Davey et al., 2016), they are unlikely to be separable in patients with stroke aphasia who typically have large lesions. Nevertheless, the extent to which semantic deficits and more general executive dysfunction co-occur varies across individuals (as reviewed by Gainotti, 2014). Taken together, these findings suggest that the major areas of lesion overlap in SA – in left inferior prefrontal and temporoparietal cortex – play a crucial role in shaping semantic retrieval to suit the demands of the task or context, accounting for the pattern of inflexible semantic retrieval that these patients show (e.g. Jefferies and Lambon Ralph, 2006; Noonan et al., 2010).

In summary, contemporary accounts of semantic cognition propose that a dynamic interplay of conceptual knowledge with control processes supports the retrieval of meaning in a manner that is tailored to the task or context (Hoffman et al., 2018; Jefferies, 2013; Lambon Ralph et al., 2016). The activation of conceptual representations is thought to be modulated by recent experience and current task goals (Yee and Thompson-Schill, 2016). As a consequence, semantic control demands should reflect the match between the semantic features required by a task and those that are most accessible for the concept (because of recent experience or the strength of long-term learning). In this way, the context in which concepts are presented will strongly influence controlled retrieval demands (Cf. Tulving and Thomson, 1973). Patients with SA provide clear evidence for this claim, since their semantic retrieval is highly sensitive to cueing. Phonological cues result in near-perfect picture naming performance in SA (but not in semantic dementia, reflecting the loss of conceptual knowledge; Jefferies et al., 2008b). Similarly, embedding an ambiguous word in a sentence that disambiguates its meaning yields a positive effect on SA patients’ performance (Noonan et al., 2010; e.g. “they served a delicious punch at the party” vs. “the boxer landed a punch on the opponent”). Picture cues are effective at supporting conceptual retrieval in non-verbal tasks: SA patients are better able to retrieve the specific action associated with a tool when shown the typical recipient of the action (Corbett et al., 2011; e.g., for hammer, a picture of a nail), in line with the proposal that their semantic control deficit is multimodal. However, sometimes concepts have to be processed in a manner that is at odds with the immediately preceding context, or the interpretation needs to change over time. In these circumstances, (mis)cues actually increase semantic control demands, since information that is irrelevant for the task (but potentially dominant for the concept) is made more accessible. SA patients show a greater cost of both phonological miscues in picture naming (Soni et al., 2009; e.g. for tiger, the phonogical cue “L”) and sentence contexts that cue the irrelevant interpretation of ambiguous words (Noonan et al., 2010; e.g., “the young men like to box " for box - packet).

Since heteromodal concepts are thought to draw on a wide range of features (cf. hub and spoke model), we would expect different kinds of cues to be effective in patients with SA. In the current study, we moved beyond the phonological and semantic cues used in previous investigations, to investigate the impact of visuo-spatial contexts and emotional cues such as facial expressions and prosody in speech. In everyday situations, patients' comprehension is likely to be supported by the environment they are in – including the location in which conceptual retrieval occurs, and the facial expression and voice intonation of speakers. However, previous studies have not examined whether SA patients rely on these kinds of cues to guide semantic retrieval. This question has become pressing, given the development of telephone and online therapy and support tools, which often lack this information. We used both valence cues (emotional faces and prosody) and pictures of the spatial context in which items commonly occur. These cue types have already been shown to be effective in episodic memory. Memory is improved when the emotional context of an encoding event is reinstated at retrieval (e.g. Bower, 1981; Bower and Mayer, 1989; Bower et al., 1978; Eich, 1995). Similarly, the spatial context in which an event is encoded appears to be an effective retrieval cue (e.g. Burgess et al., 2002; Robin et al., 2016; Robin et al., 2018; Robin and Moscovitch, 2014). However, these cue types have rarely if ever been employed in semantic retrieval tasks.

In three different experiments, we provided pictures of facial expressions, emotional prosody sequences using nonsense syllables (“ba-ba-ba”), and spatial context pictures, prior to semantic decisions in which patients were asked to match an ambiguous probe word (e.g. jam) to a semantically related target presented among distractors (e.g. jam – blanket, spoon, hospital, union). In some trials, cues were used to prime the correct interpretation of the word. In other trials, the cue was designed to activate the alternative meaning of the ambiguous word, which was not relevant for the task (miscue). We anticipated that both cueing and miscuing effects would be greater for SA patients compared to healthy controls across all tasks since (i) ventral ATL is largely undamaged in SA; consequently, the hub and spoke model envisages that diverse cues will influence the accessibility of conceptual information in the semantic store and (ii) damage to semantic control processes makes it difficult for SA patients to retrieve knowledge in the absence of external constraint, and to overcome irrelevant semantic information that is activated.

## 2 Method

### 2.1 Participants

The study was approved by the local ethical committee and informed consent was obtained. Ten SA patients were recruited through stroke and aphasia associations across Yorkshire, UK. The majority (P1-4, P6-9) have been previously described (Stampacchia et al., 2018). All patients had suffered a cerebrovascular accident (CVA) affecting the left hemisphere at least one year before testing. Background details and lesion characteristics for each patient can be found in Table 1. Consistent with previous investigations of SA, patients were selected on the basis of multimodal semantic deficits. All patients showed semantic control deficits in both verbal and non-verbal semantic tasks. They performed poorly when retrieving less-dominant meanings of homonyms in a semantic judgement task (Noonan et al., 2010) and non-canonical uses in an object use task (Corbett et al., 2011).

**Table 1:** *Quantification of lesion*: 2 = complete destruction/serious damage to cortical gray matter; 1 = partial destruction/mild damage to cortical gray matter; *Anatomical abbreviations: DLPFC*=dorsolateral prefrontal cortex; *orbIFG*=pars orbitalis in inferior frontal gyrus; *triIFG* = pars triangularis in inferior frontal gyrus; *opIFG* = pars opercularis in inferior frontal gyrus*; SMA/PMC* = supplementary morot area/pontine micturition center; *TP* = temporal pole; *STG* = superior temporal gyrus; *MTG* = middle temporal gyrus; *ITG* = inferior temporal; *FG* = fusiform gyrus; *POT* = posterior occipitotemporal area; *AG* = angular gyrus; *SMG* = supramarginal gyrus.

Case	Age	Sex	Education (leaving age)	Lesion size (%)	DLPFC		orbIFG	triIFG	opIFG	SMA/PMC	TP	STG	MTG	ITG	FG	POT	AG	SMG
					BA9	BA46	BA47	BA45	BA44	BA6	BA38	BA22	BA21	BA20	BA36	BA37	BA39	BA40
P1	60	F	18	12	1	1	–	1	1	1	1	2	–	–	–	1	1	2
P2	77	M	15	15	2	–	2	2	2	2	–	2	–	–	–	–	–	1
P3	60	F	18	12	–	–	2	1	1	2	–	2	1	–	–	2	1	2
P4	57	M	18	7	–	–	–	1	2	1	–	–	–	–	–	1	1	1
P5	71	M	18	–	–	–	2	2	2	2	–	2	2	–	–	1	1	2
P6	58	F	16	15	–	–	–	–	2	2	–	1	1	–	–	1	1	2
P7	65	M	16	14	–	–	2	1	2	2	–	2	–	–	–	1	1	1
P8	77	F	16	4	–	–	–	–	1	1	–	1	1	–	–	1	–	–
P9	39	F	16	9	–	–	–	1	2	–	–	2	–	–	–	–	–	–
P10	58	F	18	14	–	1	–	2	2	2	–	2	–	–	–	–	–	2

The SA group was compared with sixteen healthy, age and education matched control participants [mean age at recruitment: SA group = 62.2, control group = 69 years, t (24) = 1.6, p = .122; mean age when leaving education: SA group = 16.9, control group = 18.2, t (21.5) = 1.6, p = .135]. The control participants had no history of neurological or psychiatric conditions and showed unimpaired cognitive functioning on the Mini-Mental State Examination with a cut-off point of 24/30 (Folstein et al., 1975). Although the control group was on average a few years older than the SA group (but not statistically significant so), this should have worked against our hypothesis that SA show poorer semantic control.

### 2.2 Lesion analyses

**Fig. 1:**
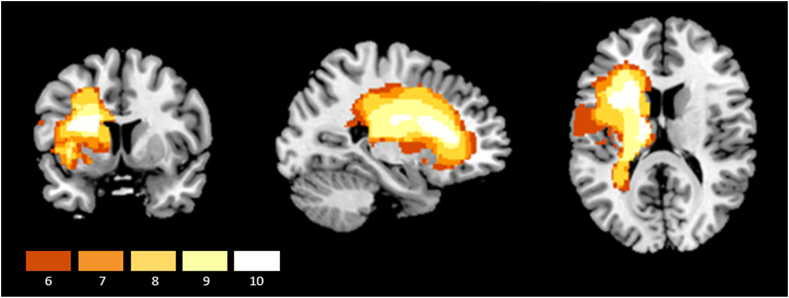
Lesion overlay of the sample of SA patients included in the study. Patients' brains were compared to aged-matched controls. Gray matter, white matter and CSF were segmented and changes from the healthy control brains were highlighted as ‘lesion’ using automated methods (Seghier et al., 2008). Only areas of maximum overlap are included (where at least 6/10 patients had a lesion). The colour bar indicates the number of patients with damage in each voxel. (For interpretation of the references to colour in this figure legend, the reader is referred to the Web version of this article.)

MRI scans were available for all 10 patients. An overlay of lesion maps was created using automated lesion identification (Seghier et al., 2008), and is displayed in Fig. 1. This technique classifies each voxel as gray matter, white matter or cerebrospinal fluid, and identifies lesions as regions of the brain that do not correspond with the expected tissue type.

Details of individual patients' lesions were obtained using Damasio's standardized templates (Damasio and Damasio, 1989) and are displayed in Table 1. All of the patients had damage within inferior frontal gyrus (IFG) (especially in pars opercularis and orbitalis). The lesion extended into superior temporal gyrus (STG) and the supplementary motor area (SMA) in the vast majority (9/10). Other areas that showed damage were supramarginal gyrus (SMG, 8/10 patients), posterior middle temporal gyrus (pMTG; 4/10 patients) and dorsolateral prefrontal cortex (DLPFC; 3/10 patients).

### 2.3 Background neuropsychological assessment

Here we briefly describe the tests used in the background assessment of our patients. This neuropsychological assessment protocol has been recently described by Stampacchia et al. (2018).

#### 2.3.1 General neuropsychology

Data for individual patients is shown in Table 2. In addition to their semantic deficits, patients often displayed more general language and executive impairments. Word repetition (PALPA 9; Kay et al., 1992) was impaired in four out of ten patients (and testing was not attempted in a further two patients because their speech production was very poor). Verbal fluency tasks (category and letter fluency) were under cut-off in seven out of ten patients and not attempted in a further three patients. The “cookie theft” picture description (Goodglass and Kaplan, 1983) revealed non-fluent speech in half of the patients. Executive and attentional impairment was observed in seven out of ten patients across four tasks: Elevator Counting with and without distraction from the Test of Everyday Attention (Robertson et al., 1994); Ravens Coloured Progressive Matrices (RCPM; Raven, 1962); Brixton Spatial Rule Attainment task (Burgess and Shallice, 1997) and Trail Making Test A & B (Reitan, 1958). This is in line with previous studies which found that deregulated semantic cognition in semantic aphasia often correlates with executive dysfunction (Jefferies and Lambon Ralph, 2006; Noonan et al., 2010). Visuo-spatial processing, as measured by the Visual Object and Space Perception Battery (Warrington and James, 1991) was spared in nine out of ten patients.

**Table 2:** Scores are number of correct; NT = unavailable for testing; NA = not attempted because patients were non-fluent. Bold underlined numbers denote impaired scores (less than two standard deviation below mean). PALPA = Psycholinguistic Assessment of Language Processing in Aphasia; TEA = Test of Everyday Attention; VOSP = Visual Object and Space Processing Battery.

Test	Max	Cut-off	Patients mean (SD)	P1	P2	P3	P4	P5	P6	P7	P8	P9	P10
*Non-semantic language tests*													
PALPA 9 real word repetition (tot.)	80	73	53.6 (32.9)	NA	**71**	**42**	79	NA	78	**1**	74	77	**7**
Category Fluency (8)	–	62	43.5 (28.8)	NA	** 26**	**15**	**26**	NA	**14**	NA	80	**57**	69
Letter Fluency (F, A, S)	–	21.8	8 (5.4)	NA	** 2**	**2**	**6**	NA	**3**	NA	**16**	**9**	**12**
Cookie theft (words/minute)	–	–	28.1 (22.3)	0	18	9	37	NA	60	0	54	37	38
*Executive and spatial processing*													
TEA: counting without distraction	7	4.2	4.6 (1.3)	**2**	5	6	NT	5	**4**	7	5	7	5
TEA: counting with distraction	10	2.6	1.9 (.9)	**1**	3	**1**	NT	**1**	**2**	7	**2**	6	**3**
Raven's coloured matrices (total)	36	28 a	29 (5.1)	31	29	31	30	**24**	**19**	34	**21**	33	33
Brixton spatial anticipation (correct)	54	28	25.8 (9.2)	**21**	**7**	**18**	**23**	34	**24**	31	31	30	**39**
Trail Making Test A (correct)	24	24 a	23.1 (1.6)	**19**	**22**	**23**	**23**	24	24	24	24	24	24
Trail Making Test B (correct)	23	17.4 a	15.5 (9.2)	**2**	23	**16**	**5**	23	**1**	23	19	22	**21**
*Visuospatial processing*													
VOSP dot counting	10	8	9.3 (1.2)	**7**	10	10	10	8	10	8	10	10	10
VOSP position discrimination	20	18	19 (1.7)	19	20	**15**	20	20	**17**	19	20	20	20
VOSP number location	10	7	8.6 (1.7)	8	10	** 5**	10	8	10	10	**5**	8	8
VOSP cube analysis	10	6	8.9 (1.1)	8	9	** 4**	9	9	7	10	10	10	**8**

a Norms from healthy controls tested at the University of York (cut-off is mean minus two standard deviation). Number of controls as follows: Ravens = 20; Trail Making Test = 14.

#### 2.3.2 Semantic memory assessment: Cambridge Semantic Battery

Individual test scores are provided in Table 3. The Cambridge Semantic Battery (Adlam et al., 2010; Bozeat et al., 2000) measures semantic retrieval for a set of 64 items across four tasks: picture naming, word-picture matching, verbal and pictorial semantic associations (Camel and Cactus Test, CCT). Patients showed large variability during picture naming [correct trials M (SD) = 62.2% (39.3)], in line with their varying degree of impairment in production, while performance was uniformly at ceiling in word-picture matching [M (SD) = 93.4% (5.9)]. When the control demands of the task were higher, such as when secondary associations between concepts were probed on the CCT in either verbal or pictorial format, patients showed greater impairment which was equivalent across modalities [words M (SD) = 80% (16.7); pictures M (SD) = 80% (15.4)].

**Table 3:** Scores are number of correct; NT = unavailable for testing; NA = testing was not attempted because patients were non-fluent. Bold underlined numbers denote impaired scores (less than two standard deviation below mean). Cut-off scores are from healthy controls tested at the University of York (mean minus two standard deviations). Number of controls as follows: Cambridge Semantic Battery = 10; Ambiguity task, Alternative object use, Synonym with distractors = 8.

Test	Max	Cut-off	Patient Mean (SD)	P1	P2	P3	P4	P5	P6	P7	P8	P9	P10
*Cambridge Semantic Battery*													
Picture Naming	64	59.1	39.8 (25.1)	**1**	61	**19**	**50**	**0**	60	**3**	**56**	62	**46**
Word-Picture Matching	64	62.7	59.8 (3.8)	63	**62**	**60**	**62**	**56**	**62**	**52**	**56**	**62**	63
Word CCT	64	56.6	51.2 (10.7)	**39**	**43**	**29**	**52**	**56**	59	57	61	60	**56**
Picture CCT	64	52.7	51.2 (9.8)	**31**	**44**	**45**	57	61	**45**	54	53	61	61
*Ambiguity task*													
Miscued dominant	30	30	19.3 (5.6)	**12**	**13**	**13**	**19**	NT	**20**	**21**	**24**	**26**	**26**
Miscued subordinate	30	26.6	15.4 (6.3)	**7**	**10**	**14**	**15**	NT	**10**	**18**	**18**	**19**	28
No cue dominant	30	28.4	24.9 (3.1)	**22**	**18**	**24**	**26**	**25**	**24**	**27**	**28**	**28**	**27**
No cue subordinate	30	27.6	16.6 (4.1)	**11**	**9**	**14**	**17**	**16**	**19**	**19**	**21**	**19**	**21**
Cued dominant	30	30	24.2 (3.5)	**23**	**21**	**19**	**23**	NT	**24**	**23**	**27**	**29**	**29**
Cued subordinate	30	28.8	22.9 (4.6)	**25**	**14**	**20**	**28**	NT	**19**	**24**	**23**	**25**	**28**
*Synonym with distractors*													
Strong	42	35.4	20.1 (8.1)	**15**	**12**	**13**	**23**	**16**	**21**	**30**	**22**	**17**	38
Weak	42	40.4	30 (4.9)	**25**	**23**	**29**	**30**	**33**	**27**	**31**	**28**	**39**	**36**
*Object use*													
Alternative	37	33.9 ^a^	22.8 (7.5)	**14**	**13**	**14**	**22**	**22**	34	**22**	**26**	**29**	**32**
Canonical	37	n.a	34.3 (2.9)	32	31	29	35	35	37	33	37	37	37

#### 2.3.3 Tests of semantic control

In line with the inclusion criteria adopted in previous studies by our group (e.g., Stampacchia et al., 2018) the patients in this study had difficulties in retrieving and manipulating concepts in a flexible manner, due to deficient semantic control processes. We report their performance on three tasks that manipulated the control demands of verbal and non-verbal semantic judgements. The task descriptions are taken from Stampacchia et al. (2018) and therefore appear in quotation marks. Individual test scores are displayed in Table 3.

i. *Ambiguity task* (Noonan et al., 2010)*.* “Semantic judgements (60 items) probed the dominant (money) and subordinate (river) meanings of ambiguous words (e.g., bank). These semantic decisions were uncued or preceded by a sentence that primed the relevant meaning (cue condition e.g., for money, i went to see the bank manager) or irrelevant interpretation (miscue condition e.g., the bank was slippery). There were four response options on each trial.” All patients, with the exception of P5 who only completed the no cue condition, were below the normal cut-off in all conditions. They showed better comprehension for dominant than for subordinate interpretations [no cue condition accuracy: dominant M (SD): 83% (10.4); subordinate M (SD) = 55.3% (13.7)] and had greater difficulties in accessing subordinate meanings following miscues rather than cues [subordinate trials: miscues M (SD) = 51.5% (21); cues M (SD) = 76.3% (15.1)].
ii. *Synonym judgment task.* “We tested synonym judgement with strong or weak distractors (84 trials), using a task from Samson et al. (2007); e.g., dot with point [target], presented with dash [strong distractor] or leg [weak distractor]. There were three response options per trial.” Accuracy was below the cut-off for all patients, with the exception of P5 who did not take part and P10 who scored above the cut-off in the strong distractor condition. Performance was poorer when semantically-related but irrelevant distractors were presented [t (9) = 4, p = .003].
iii. *Object use task.* “The object use task (74 items), from Corbett et al. (2011), involved selecting an object to accomplish a task (e.g., bash a nail into wood), with all items represented as photographs. The target was either a canonical tool, normally used to complete the task (e.g., hammer), or an alternative non-canonical option (e.g., brick), presented among a set of five unsuitable distractors.” Patients were poorer at selecting non-canonical than canonical targets [t (9) = 7.2, p < .001]. One patient (P6) was not below the normal cut-off in the non-canonical condition.

In summary, all ten patients showed impaired performance on one or more non-semantic verbal tasks, while they showed impaired performance on all semantic tasks. The SA group exhibited strong sensitivity to manipulations of semantic control demands across modalities – i.e., more impaired comprehension of subordinate than dominant interpretations of ambiguous words; sensitivity to cues and miscues; better performance with weak than strong distractors and better retrieval of canonical than alternative object use. A composite score reflecting each patient's deficits in semantic cognition was derived from the Cambridge Semantic Battery and the three semantic control tasks described above using factor analysis. Patients are ordered by this composite score in the tables below.

## 3 Multimodal cueing paradigms

Three experiments investigated the effects of cues and meaning dominance on semantic judgements. Trials could be cued, miscued, or presented without a cue. The probe word that followed the cue was always an ambiguous word with more than one meaning. In half of the trials, the dominant meaning of the word was probed (e.g. bank-money), while the remaining trials referred to the subordinate meaning (e.g. bank-river). Given the multimodal nature of semantic cognition, we investigated whether both modality (for visual vs. auditory emotional cues) and informational content (visuo-spatial vs. emotional) would prime concepts in a similar way. We addressed this question in three separate experiments. In the first, we used facial emotional expressions as cues and miscues – these were consistent or inconsistent with the valence of the ambiguous word that was relevant in the subsequent semantic decision. In the second experiment, we used prosody within short ‘*ba-ba-ba*’ sequences spoken in different emotional tones (e.g., happy or sad voices), which were again consistent or inconsistent with the valence of task-relevant interpretations of the ambiguous words. Finally, in the third experiment, we provided participants with photographs of visuo-spatial scenes: these either cued the relevant interpretation or miscued the irrelevant interpretation of the ambiguous words. The materials and experimental procedure were similar across the three experiments. A thorough description of the methods is provided only for Experiment 1, while for Experiments 2 and 3 we highlight any differences with the original protocol.

### 3.1 Experiment 1. facial emotional expressions

#### 3.1.1 Materials

Forty-three ambiguous probe words were selected using published word norms. Thirty-four were selected from the University of Alberta norms of relative meaning frequency (Twilley et al., 1994). In half of the trials, the probe was used in its *dominant* meaning, while in the remaining trials the *subordinate* meaning of the word had to be retrieved. For five additional words, only the dominant meaning was listed; the subordinate meaning was presumed to be rarer. Four remaining words were assigned to the dominant/subordinate conditions using Edinburgh Associative Thesaurus (Kiss et al., 1973). Whenever possible, we chose meanings with different emotional valence (e.g. strawberry jam is typically thought to be nice, whereas traffic jams are normally associated with negative emotions). Target words for the dominant and the subordinate interpretations were matched for lexical frequency (CELEX database; Baayen et al., 1993) (t (84) = 0.1, p = .887), length (t (84) = 0.4, p = .680), number of syllables (t (84) = 0.3, p = .774) and imageability (t (84) = 0.6, p = .571) in the N-Watch database (Davis, 2005). Each probe was presented alongside four alternatives, namely a semantically related target and three semantically unrelated distractors.

**Fig. 2:**
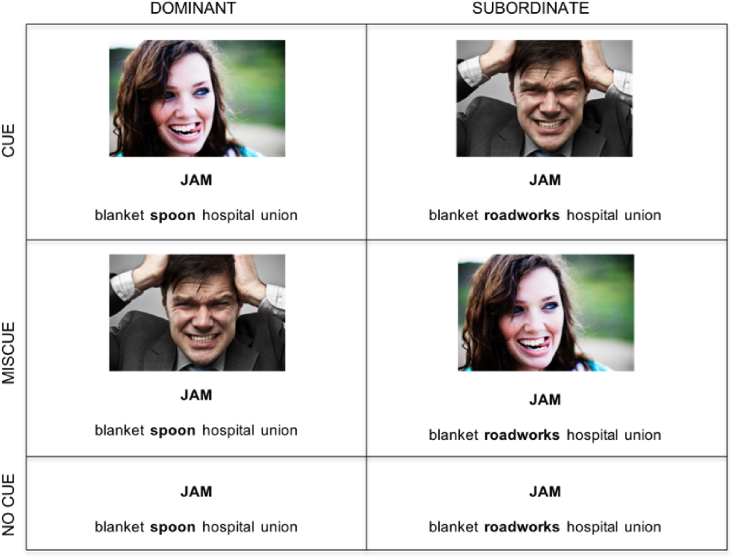
The 6 possible combinations of *cue condition* and *dominance* for the probe word “Jam” are shown here.

We manipulated the control demands of the task by showing facial expressions that were either consistent with the relevant interpretation of meaning (*cue condition* - e.g./happy face/jam [jelly]), or with the alternative and therefore irrelevant interpretation (*miscue condition* - e.g./angry face/jam [traffic jam]). The same image was used as a cue in one trial, and as a miscue in another trial. In one third of the trials, the probe was presented in the absence of a cue (*no cue condition*). Images included the eight basic emotions from the Radboud Faces Database (Langner et al., 2010): happy, angry, sad, contemptuous, disgusted, neutral, fearful, surprised. These were supplemented with images of more nuanced emotional expressions (see Fig. 2). These images only included the face and shoulders on a neutral background. Participants saw each probe word 6 times, once in each combination of cue-condition/meaning dominance (see Fig. 2). Target words also appeared as distracters on a different trial. After the experiment, we asked control subjects to judge the valence of these words (e.g. “do they leave you with either good or bad feelings?”) on a scale from 0 (not at all) to 7 (very much). Ratings were collected for the probes presented alone, as well as for each probe-target combination. This allowed us to remove any non-emotional pairings.

#### 3.1.2 Procedure

The experiment was run using E-Prime v1.1 (Schneider et al., 2002). Before the beginning of each block, patients received verbal and written instructions about the nature of the task, while healthy controls received written instructions only. On any given trial, a probe word was presented for 2 s (alongside an image of a facial expression in the *cue* and *miscue* conditions), then the target and three distracters appeared in written format below the probe. These were read aloud by the experimenter to facilitate patients' comprehension. Participants had 10 s to respond, before the next trial was presented and an error was recorded. As most of the patients had motor impairments at the time of testing, patients gave their response by pointing to one of the options and the experimenter pressed the corresponding key on their behalf. All participants had 10 s to respond before the next trial was presented and an error was recorded. Accuracy and response time (RT) were recorded on each trial. Multiple researchers were involved in collecting this data but the conditions were counterbalanced across sessions in each experiment, reducing the impact of variability in the way RT was recorded. Moreover, the experimenter maintained one finger on each of the four possible keys to minimize the time between the patients’ decision and the actual keypress.

Two practice items were presented before the start of each block. A total of 258 trials were arranged in 6 blocks of 43 trials each, with each probe used once per block. Block order was counterbalanced across participants, and trial order was randomized to control for possible effects of the order of presentation. Within a session, both meanings of the probe word were primed. Cue type was counterbalanced, such that a roughly equal number of cue/miscue/no cue trials appeared in each block. At the end of the experiment, control participants rated the stimuli for how emotive they were (see Materials section).

The responses to seven ambiguous words were removed from the main analysis, due to consistently poor performance on those trials in the control participants. We identified items for removal by collapsing accuracy data across cueing conditions and obtaining average scores for dominant and subordinate trials. Ambiguous words which did not have both dominant and subordinate average scores above 50% accuracy in control participants were not carried forward into the analyses. This brought the number of ambiguous words in each of the six conditions to 36.

#### 3.1.3 Statistical analyses

At the group level, accuracy and response efficiency (median RT/mean accuracy) data were analyzed separately using three-way mixed ANOVAs, with cue condition (3 levels: cue, miscue, no cue) and dominance (2 levels: dominant, subordinate) as within-subjects factors, and group (2 levels: controls, patients) as a between-subjects factor. Pairwise comparisons for all significant interactions were Bonferroni-corrected. All statistical analyses were performed in SPSS version 24 (IBM, Armonk, NY).

#### 3.1.4 Results

**Fig. 3:**
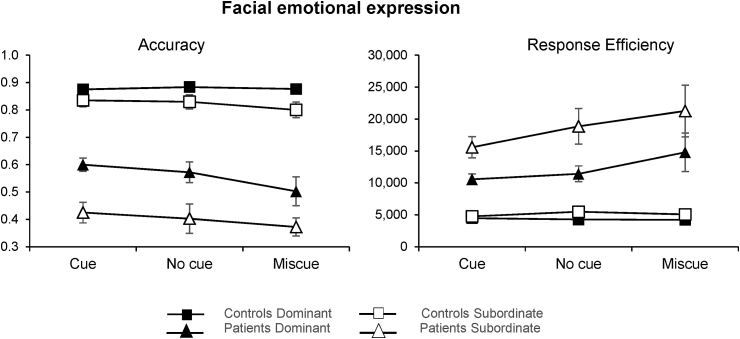
Mean accuracy (left) and median response efficiency (right) for patients and controls in the six different combinations of cue condition and dominance. Small numbers indicate poorer performance in the accuracy graph (left), while they reflect better performance when expressed as response efficiency (right). Error bars show Standard Error of the Mean (SEM).

Mean accuracy and median response efficiency are displayed in Fig. 3. ANOVA results are reported in Table 4. In Experiment 1, there were main effects of group, cue type, and ambiguity plus two-way interactions of group with cue type (significant only in response efficiency) and group with ambiguity. Overall, patients were less accurate and less efficient than controls. Bonferroni-corrected pairwise comparisons of the group by cue type interaction indicated that patients’ efficiency on miscue trials was significantly lower compared to cue trials (p = .002), while the same was not true for healthy controls (p = 1). Performance was also less accurate and less efficient for the subordinate meaning of the ambiguous word. Post-hoc tests of accuracy data revealed that both groups were less accurate when retrieving subordinate meanings (patients: p < .001; controls: p = .002). This effect was greater in the patients, in line with the expected pattern of deregulated semantic control in SA (see Fig. 3). Patients were also less efficient on subordinate compared to dominant trials (p < .001), while controls did not show a significant difference (p = .417).

### 3.2 Experiment 2. emotional prosody

#### 3.2.1 Materials

While in the previous task we presented participants with visual emotional cues (faces), here we used sound cues featuring different emotions. These consisted of simple monosyllabic sounds spoken with emotional prosody. Twenty-four items were recorded from either a male or female voice repeating ‘ba-ba-ba-ba’ sounds in a way that reflected a variety of emotions (e.g. happy, irritated, surprised), as rated by control participants after the experiment. The stimuli lasted between 2 and 3 s and background noise was removed using Audacity software (ver. 2.1.2; Mazzoni and Dannenberg, 2000). The same set of ambiguous words presented in Experiment 1 was used.

#### 3.2.2 Procedure

At the beginning of each trial, an ambiguous word appeared in the middle of the screen. Participants were instructed to press the spacebar to hear the cue sound, which could be either emotionally congruent or incongruent with the relevant interpretation of the ambiguous word. At the offset of the sound, the four options were presented below the probe. As before, the task was to select the word that was semantically related to the probe, while discarding the three distracters. There were four blocks, containing 172 trials. As the word stimuli were identical to those in Experiment 1, data for the no cue condition were taken from this experiment. Ambiguous words with an average of <50% accuracy for controls across cue, no cue and miscue conditions were removed from the analysis, bringing the number of ambiguous words in each of the 4 conditions to 36.

#### 3.2.3 Results

**Fig. 4:**
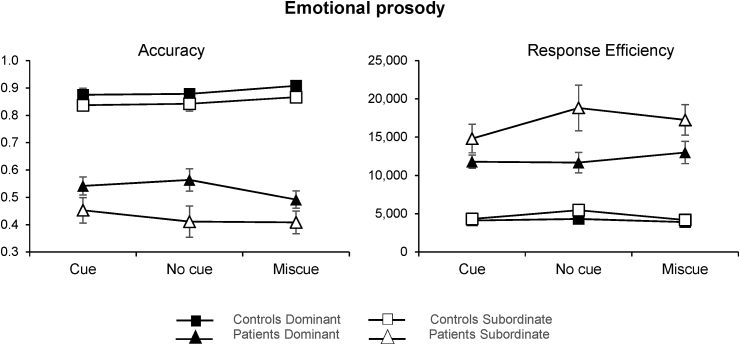
Mean accuracy (left) and median response efficiency (right) for patients and controls in the six different combinations of cue condition and dominance. Small numbers indicate poorer performance in the accuracy graph (left), while they reflect better performance when expressed as response efficiency (right). Error bars show SEM.

Mean accuracy and median response efficiency are displayed in Fig. 4. ANOVA values are reported in Table 1. There were significant main effects of group and ambiguity in both accuracy and response efficiency, while the effect of cue condition approached significance (p = .057) in response efficiency. There were two-way interactions between group and dominance, and cue type by dominance (this last one being significant only in response efficiency). The interaction between group and cue approached significance in the accuracy data (p = .058). Performance was poorer when the task required participants to retrieve the subordinate meaning of the ambiguous word. As in experiment 1, both groups were less accurate with subordinate meanings (patients: p < .001; controls: p = .009), with the patients showing a stronger effect, but only the SA group had lower efficiency on subordinate vs. dominant interpretations (patients: p < .001; controls: p = .317). Moreover, pairwise comparisons of the cue type by dominance interaction revealed that the ambiguity effect in the patient group was greater in the absence of a cue, such that patients were less efficient at retrieving the subordinate meaning of a word when no cue was provided (p = .016). The same was not true for controls (p = 1).

### 3.3 Experiment 3. visuo-spatial context

#### 3.3.1 Materials

**Fig. 5:**
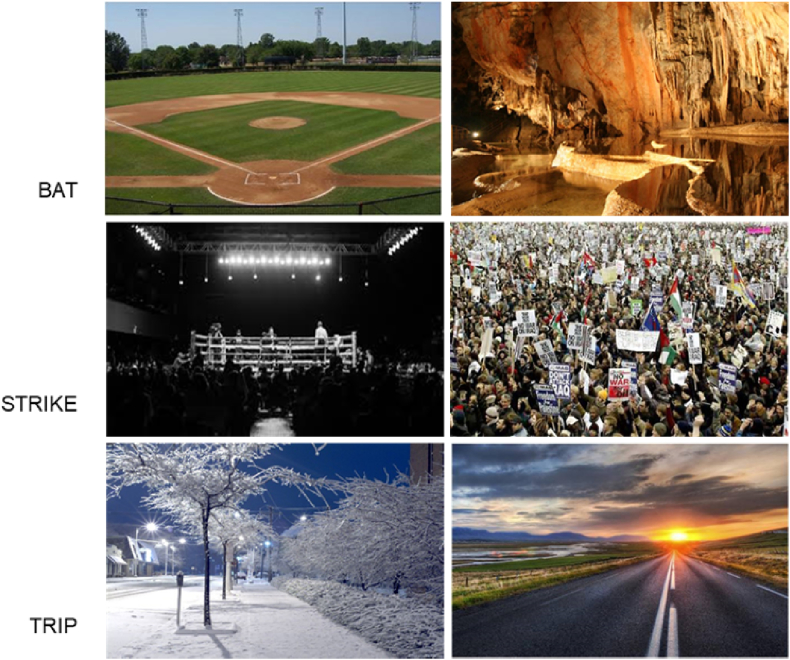
Location cues for three probes words used in the dominant meaning (left) and in the subordinate meaning (right). From top to bottom: BAT-team/BAT – night; STRIKE – bruise/STRIKE – union; TRIP – balance/TRIP - car.

Here, the cue consisted of a visuo-spatial context, rather than an emotional one. Stimuli were photographs of scenes (Fig. 5) linked to either the relevant meaning (*cue condition*) or an alternative interpretation (*miscue condition*) of an ambiguous word. For example, the cue for BAT-team could be a picture of a baseball field, whilst BAT-night could be an image of a cave. Forty-five ambiguous words were used, of which fifteen were also presented in Experiment 1 and 2. Of the remaining, twenty-seven were taken from Elston-Gúttler and Friederici (2005) and three from the Edinburgh Association Thesaurus (Kiss et al., 1973). Target words for the dominant and the subordinate interpretations were matched for lexical frequency (CELEX database; Baayen et al., 1993) (t (88) = 0.6, p = .532), length (t (88) = 0.2, p = .799), syllable length (t (74) = 1.8, p = .080) and imageability (t (88) = 0.4, p = .704) using the N-Watch (Davis, 2005).

#### 3.3.2 Procedure

The procedure followed Experiment 1 and 2. A visuo-spatial scene was presented simultaneously with the ambiguous probe for 2 s. At the end of this period, four options appeared below. The participants’ task was again to select the semantically associated word while discarding the distracters. Trials in which controls had poor accuracy were removed, as in Experiment 1 and 2. Of the original 45 ambiguous words presented in each condition, 36 were carried forward into the analyses.

#### 3.3.3 Results

**Fig. 6:**
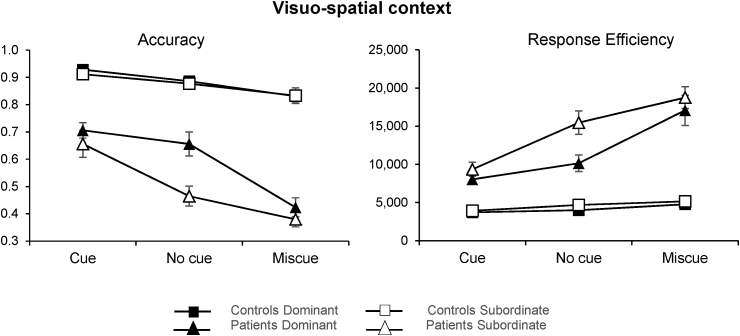
Mean accuracy (left) and median response efficiency (right) for patients and controls in the six different combinations of cue condition and dominance. Small numbers indicate poorer performance in the accuracy graph (left), while they reflect better performance when expressed as response efficiency (right). Error bars show SEM.

Mean accuracy and median response efficiency are displayed in Fig. 6. ANOVA values are reported in Table 4. We found a three-way interaction between group, dominance, and cue type. Separate ANOVAs were conducted for accuracy and response efficiency in the patients and in the control group. We found a significant interaction between cue condition and dominance in the patient group, in both accuracy (F (2, 18) = 8.9, p = .002) and median response efficiency (F (2, 18) = 4, p = .036), but no interaction in the control group. Bonferroni-corrected comparisons of accuracy in the patient group revealed more errors for miscues compared to both cues (t (8) = −9.1, p < .001) and the no cue condition (t (8) = −8.9, p < .001) for the dominant interpretation. When the subordinate meaning was required, the provision of a cue significantly improved accuracy relative to the miscue (t (8) = 4.4, p = .005) and no cue (t (8) = 4.7, p = .004) conditions. The same pattern of results was obtained for response efficiency: for the dominant interpretation, patients were impaired by miscues relative to cues (t (8) = 5.9, p = 001) and no cue trials (t (8) = 5.5, p = 001), while for the subordinate meaning, the same positive effect of cueing compared to miscues (t (8) = −5.7, p = .001) and no cue (t (8) = −4.8, p= .003) was obtained.

**Table 4:** Accuracy and response efficiency effects revealed by three-way mixed ANOVAs of the data for Experiments 1, 2, and 3. Significant results and interactions are reported in bold and marked with *. A Greenhouse-Geisser correction was applied where the assumption of sphericity was not met.

			Group	Cue Condition	Dominance	Cue condition x Group	Dominance x Group	Cue condition x Dominance	Cue condition x Dominance * Group
FACIAL EMOTIONS	Accuracy	F	140.9*	3.8*	68.0*	1.4	15.2*	0.0	1.3
		df	1, 24	2, 48	1, 24	2, 48	1, 24	2, 48	2, 48
		p	<.001	.029	<.001	.249	.001	.953	.296
		partial η2	0.9	0.1	0.7	0.1	0.4	0.0	0.0
	Response Efficiency	F	53.4*	5.7*	21.4*	5.7*	13.0*	0.7	0.1
		df	1, 24	2,48	1, 24	2,48	1,24	1.3, 30.0	2,48
		p	<.001	.006	<.001	.006	.001	.426	0.862
		partial η2	0.7	0.2	0.5	0.2	0.4	0.0	0.0
PROSODY	Accuracy	F	146.2*	0.1	44.3*	3.0	10.0*	1.3	1.6
		df	1, 24	2,48	1, 24	2,48	1, 24	2,48	2,48
		p	<.001	.894	<.001	.058	.004	.284	.210
		partial η2	0.9	0.0	0.6	0.1	0.3	0.1	0.1
	Response Efficiency	F	68.7*	3.0	39.5*	1.8	25.2*	5.3*	2.0
		df	1, 24	2,48	1, 24	2,48	1, 24	2,48	2,48
		p	<.001	.057	<.001	.182	<.001	.009	.153
		partial η2	0.7	0.1	0.6	0.1	0.5	0.2	0.1
VISUO-SPATIAL	Accuracy	F	144.7*	62.8*	17.8*	17.0*	12.7*	6.9*	6.5*
		df	1, 24	1.6, 38.3	1, 24	2, 48	1, 24	2, 48	2, 48
		p	<.001	<.001	<.001	<.001	.002	.002	.003
		partial η2	0.9	0.7	0.4	0.4	0.3	0.2	0.2
	Response Efficiency	F	109.2*	61.2*	24.7*	37.2*	13.2*	7.5*	4.9*
		df	1, 24	2,48	1, 24	2,48	1, 24	2,48	2,48
		p	<.001	<.001	<.001	<.001	.001	.001	.012
		partial η2	0.8	0.7	0.5	0.6	0.4	0.2	0.2

A Cochran's Q test was used to compare the three levels of *cueing* at the individual level. This test revealed that 7 out of 10 patients showed a significant difference between the three cue conditions (p = .010 to p < .001).

## 4 Discussion

This study explored the effect of multi-modal cues on conceptual tasks in SA patients with deregulated retrieval following left-hemisphere stroke. Across three experiments we presented emotional facial expressions (Experiment 1), emotional prosody (Experiment 2) and visuo-spatial contexts (Experiment 3), which were designed to cue or miscue the currently-relevant or irrelevant interpretations of ambiguous words. SA patients were highly sensitive to these cues, showing better performance when external information was consistent with semantic knowledge to be retrieved, and poorer performance when the cue was misleading. Both emotional and visuo-spatial cues were effective.

Previous studies by our group have shown that patients with SA are highly influenced by semantic ambiguity, with poorer performance when the task requires the less dominant interpretation of the word (Noonan et al., 2010). Across all three experiments we replicated this effect of ambiguity in an independent sample. In line with previous findings of cueing and miscuing effects, performance was modulated in both positive and negative directions by the provision of information that was relevant or irrelevant to the task. However, we used emotional and spatial cues, which have not been previously investigated. Since heteromodal concepts are thought to draw on a wide range of features, we expected different kinds of cues to be equally effective in patients with SA. Perhaps not surprisingly, the strongest effect of cueing was observed in Experiment 3. Visuo-spatial contexts are likely to provide a highly concrete and vivid interpretation of the word, constraining semantic retrieval to a large extent. Emotional cues also influenced performance, at least when presented using facial expressions (Experiment 1). On the other hand, the effect of cueing in the emotional prosody task (Experiment 2) only approached significance. Facial expressions might be stronger cues to emotion than prosody. Nevertheless, given the known right-hemisphere dominance for emotional processing (as reviewed by Gainotti, 2019), we expected our left-hemisphere stroke patients to be able to extract the valence of the emotional stimuli, regardless of the modality of presentation (visual vs. auditory).

Our results are consistent with contemporary accounts of semantic cognition such as the Controlled Semantic Cognition account (Jefferies, 2013; Lambon Ralph et al., 2016), which anticipates interactions between semantic representations and control processes in conceptual retrieval. This framework proposes a ‘graded hub’ for conceptual representation in ventral ATL – an area relatively invulnerable to stroke, and largely spared in SA patients. This region is thought to allow the computation of coherent conceptual representations from combinations of diverse features – including valence and visuospatial context, as well as visual and auditory inputs. While there is most evidence for the graded combination of vision and audition, recent work has suggested that the ATL hub region integrates emotional valence (Olson et al., 2013; Ross and Olson, 2010), via connections from orbitofrontal cortex via the uncinate fasciculus (Highley et al., 2002; Papinutto et al., 2016; Von Der Heide et al., 2013 and Olson et al., 2013). Several studies have shown an involvement of portions of the ATL in representing and retrieving social knowledge (Binney et al., 2016; Olson et al., 2013; Rice et al., 2018; Ross and Olson, 2010). Representations capturing spatial context within the medio-temporal complex (Bicanski and Burgess, 2018; Burgess, 2002; Burgess et al., 2002), are also likely to contribute to conceptual processing in ventral ATL, with bidirectional connections via the entorhinal cortex (e.g. Squire and Zola-Morgan, 1991). The ventral ATL is equidistant from all these diverse inputs along the cortical surface, and this is thought to facilitate the formation of heteromodal concepts (Lambon Ralph et al., 2016; Margulies et al., 2016; Visser et al., 2010; Visser and Lambon Ralph, 2011).

The graded hub account predicts that these inputs to ventral ATL can be potent cues or miscues, depending on whether they are consistent or inconsistent with current task demands. Consequently, cues that increase the accessibility of task-relevant features reduce semantic control demands, while miscues that increase the accessibility of task-irrelevant features increase semantic control demands. Here we provide further evidence for this theoretical framework by showing that emotional and spatial cues modulate the accessibility of semantic representations. Spatial context is known to play a key role in episodic memory (e.g. Burgess et al., 2001; Burgess et al., 2002; Hazen and Volk-hudson, 2018; O’keefe and Nadel, 1978; Robin et al., 2018, 2016). Similarly, emotional cues have been shown to be powerful cues in episodic memory (as reviewed by Buchanan, 2007). For example, mood induction and mood congruency paradigms have provided strong evidence for the idea that episodic retrieval is improved when there is emotional congruency between encoding and retrieval (Bower and Mayer, 1989; Bower et al., 1978; Robinson and Rollings, 2011; Xie and Zhang, 2018). At present, the contribution of these feature types to semantic retrieval has been little investigated. A key contribution of the current study is to show that these features are effective cues and miscues, particularly in people with a reduced capacity to internally constrain their semantic retrieval. However, as the multimodal cueing paradigm implemented here has not been used before, replicating the effects in a larger sample will help to clarify their magnitude, and whether spatial cues and facial expressions are more potent than emotional prosody.

The semantic control regions typically damaged in SA are spatially distinct from, but adjacent to, multiple-demand regions that support domain-general cognitive control (Davey et al., 2016; Noonan et al., 2013b). Patients with SA have large lesions, and domain-general control and semantic control networks are likely to be damaged together. Patients with SA have a broad range of deficits, as observed by Head and Luria in their seminal characterizations of the syndrome (Head, 1926; Luria, 1973). In our sample, neuropsychological tests show that 9/10 patients have some degree of executive impairment, mirroring the initial results of Jefferies and Lambon Ralph (2006) who studied an independent sample of SA cases. Given these considerations, we cannot conclude that increased sensitivity to cues in semantic tasks specifically reflect semantic control deficits in SA – this pattern may also reflect the influence of domain-general executive deficits.

The observation that semantic aphasia patients are sensitive to emotional and spatial cues is relevant to clinical practice and patient management. Showing that semantic retrieval can be influenced by emotions and spatial contexts in a semantic task might provide an explanation for why patients with SA appear to function well in everyday contexts. Real-world situations are generally very rich and characterized by both emotional and spatial cues, which can support comprehension when they are coherent with the message being communicated. Moreover, our findings suggest that patients will be vulnerable to being misled by emotional expressions and spatial context when these are not consistent with the information required in a certain situation. For example, they might be more likely to be confused when sad news is conveyed with a smile, or when a familiar object has to be used in a novel spatial context. Being aware that patients rely on contextual cues but can also be misled by them has important implications for patients, their families and therapists, since the context in which semantic retrieval occurs can be controlled to afford good understanding. A final consideration is that real-world situations are much richer than any experimental tasks designed to investigate semantic retrieval. Further research is required to investigate the potential additive effects of cues, as well as the efficacy of more ecological cues, closer to every-day situations.

## CRediT authorship contribution statement

**Lucilla Lanzoni:** Validation, Formal analysis, Data curation, Writing - original draft, Writing - review & editing, Visualization. **Hannah Thompson:** Conceptualization, Methodology, Software, Resources, Supervision, Project administration. **Danai Beintari:** Conceptualization, Methodology, Investigation, Data curation. **Katrina Berwick:** Conceptualization, Methodology, Investigation, Data curation. **Harriet Demnitz-King:** Conceptualization, Methodology, Investigation, Data curation. **Hannah Raspin:** Conceptualization, Methodology, Investigation, Data curation. **Maria Taha:** Conceptualization, Methodology, Investigation, Data curation. **Sara Stampacchia:** Resources, Investigation. **Jonathan Smallwood:** Conceptualization, Writing - review & editing. **Elizabeth Jefferies:** Conceptualization, Supervision, Funding acquisition, Writing - review & editing.
