# Corrigendum to “Emotion and location cues bias conceptual retrieval in people with deficient semantic control” [Neuropsychologia 131 (2019) 294–305]

**DOI:** 10.1016/j.neuropsychologia.2020.107739

**Published:** 2021-02-12

**Authors:** Lucilla Lanzoni, Hannah Thompson, Danai Beintari, Katrina Berwick, Harriet Demnitz-King, Hannah Raspin, Maria Taha, Sara Stampacchia, Jonathan Smallwood, Elizabeth Jefferies

**Affiliations:** a Department of Psychology, University of York, UK; b School of Psychology, University of Surrey, UK; c Faculty of Brain Sciences, University College London, UK

The authors regret to note that, on page 300 section 3.2.3, line 16–19, the text “revealed that the ambiguity effect in the patient group was greater in the absence of a cue, such that patients were less efficient at retrieving the subordinate meaning of a word when no cue was provided (p = .016). The same was not true for controls (p = 1).“, should instead appear as “revealed that people were more efficient at retrieving the subordinate meaning of words following a cue compared to when no cue was provided (p = .016). The same was not true for dominant trials (p = 1).”

The authors would like to apologise for any inconvenience caused.

